# Ultra-thin inorganic oxide and nitride films as an alternative blocking layer for electrochemical sensors

**DOI:** 10.1016/j.tsf.2025.140712

**Published:** 2025-06-10

**Authors:** Adam McHenry, Grant Jolly, Thomas Young, Michael Brothers, Victoria Coyle, Steve Kim, Jason Heikenfeld

**Affiliations:** a University of Cincinnati, College of Engineering and Applied Sciences, Biomedical Engineering, Cincinnati, OH, 45221, USA; b 711th Human Performance Wing, Air Force Research Laboratory, Wright-Patterson AFB, OH, 45433, USA; c AeroVironment Inc., 4401 Dayton-Xenia Road, Dayton, OH, 45432, USA

**Keywords:** Electrochemical sensor, Dielectric, Blocking layer, Biosensor, Aptamer

## Abstract

The desire to translate biosensors for real time molecular monitoring has intensified due to the commercial success of 2-week continuous glucose monitors. However, a common limitation for emerging biosensors is that their lifetimes are often too short for commercially expected benchmarks of at least 3-day and ideally 2-week operation. Electrochemical sensors remain the preferred format of biochemical sensing thanks to their low cost, size, weight, and power requirements for mobile deployment. When exposed to biological fluid, all electrochemical sensors require a blocking layer to protect the electrode surface from fouling and redox interferents. Traditional blocking layer approaches rely on self-assembled monolayers which are often fragile to biological interferents like proteins and require specific electrode materials to improve their stability. Presented here is an evaluation of ultra-thin inorganic oxide and nitride films as an alternative to self-assembled monolayer blocking layers. Specifically, silicon oxide, silicon nitride, and aluminum oxide films were deposited by electron beam evaporation or atomic layer deposition at thicknesses of several nanometers to mimic the electrical capacitance of a conventional monolayer blocking layer. These oxide films were characterized over 7-days and demonstrated to provide poor protection against interfering redox currents from dissolved ferricyanide (150 – 300 μA/cm2) and oxygen reduction interference (30 – 60 μA/cm2). The oxide films were then used as a blocking layer in an electrochemical aptamer sensor using the previously published aptamer for phenylalanine. The phenylalanine sensor showed a binding affinity stronger than found in literature, but a reduced signal gain (~ 20 % change in methylene blue redox current compared to the expected 50 % previously published on gold). It is speculated and supported by literature that these oxide and nitride films gradually dissolve over periods of days in an aqueous environment. Results further show that if lower quality oxide or nitride films are used, they may be more stable, but at the cost of initially higher in currents. While oxide and nitride films fail to improve upon the performance of thiol-blocking layers on gold electrodes, they may provide utility in some applications by allowing for alternate electrode materials and surfaces to be used instead of traditional self-assembled monolayers on gold electrodes.

## 1. Introduction

Real time measurement of redox reactions is not only a fundamental building block of electrochemistry but has emerged as a critical tool in the development of electrochemical sensors [1]. Longitudinal measurement (days to weeks) of analytes requires inert electrode materials that have reduced susceptibility to interferents in the solution. A highly challenging application is biosensors, where the working electrode surface must resist fouling by solutes and suppress background current, such as oxygen reduction current. Commercial biosensors such as continuous glucose monitors have set a very high bar for reliability at two weeks of continuous, accurate, and calibration free operation *in-vivo* [2]. While enzymatic sensors are the only commercial technology currently available for continuous molecular monitoring, electrochemical aptamer sensors are arguably a broader platform technology as they can be rapidly and inexpensively discovered for more analytes of interest than enzymatic sensors. However, aptamer sensors suffer critically from degradation during longitudinal measurements [3,4].

**Fig. 1.**
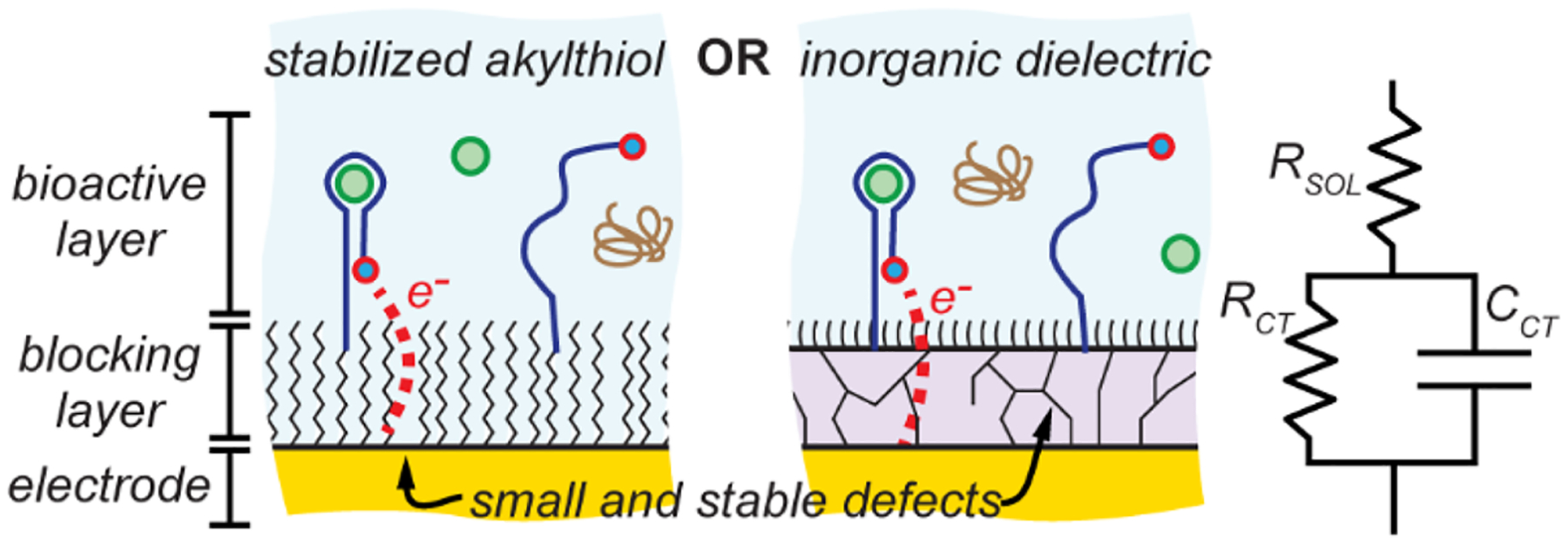
Comparison between traditional alkyl thiol monolayer (left) and the proposed oxide or nitride blocking layers (right). Both feature similar arrangements and can be modeled using a Randle’s equivalent circuit.

Electrochemical aptamer sensors use redox tagged nucleotide sequences. Most aptamer sensor demonstrations rely on self-assembled thiol attachment chemistry for both aptamer and blocking layer functionalization (Fig. 1, left). The blocking layer reduces fouling and background currents [5,6]. Thiol-based monolayers have historically exhibited limited stability *in-vivo* and *in-vitro* (hours) due to thiol oxidation, electrochemical and thermal desorption and protein interference from biological fluids [7–9].

Recently, multiple groups have demonstrated improved durability of electrochemical aptamer bound (EAB) sensors using alternative scanning methods, longer alkylthiol monolayers, and monolayer optimization [10–13]. Our research group recently reported > 1 week durability of operation of such monolayers using electrochemical potential to suppress thiol-oxidation along with extended carbon chains to increase Van der Waals interaction between adjacent blocking layer molecules [12]. These thiol monolayers with greater stability are highly dependent on gold surface roughness which can complicate electrode fabrication [12,14]. Therefore, simpler to implement electrodes are potentially desired that can be placed onto a larger variety of surfaces, metals, and semiconductors. Alternatives to thiol on gold monolayers are also therefore worth exploring, especially for long-term applications such as multi-month or multi-year implanted sensors. Considerations for a blocking layer include the following:

1. Blocking layer density: the blocking layer should limit diffusion of common redox active interferents, like oxygen, to the electrode.
2. Surface chemistry: The ability to incorporate protective antifouling chemistry to prevent foulant build up which can hinder sensor performance [12].
3. Low capacitance: Charging and discharging of the capacitive double layer should be fast enough to enable rapid voltametric techniques in the millisecond time regime [13].
4. Electrode compatibility: The ability to deposit on electrode materials other than gold enabling broader applications and device formats [15].
5. Easy Fabrication: Simple deposition methods that are fast, precise, economical, and industrially scalable for translation to real-world devices.

Other researchers have explored systems to achieve alternates to thiol monolayers on gold, including self-assembled monolayer formation on conductive oxides or carbon materials [15,16]. The challenge here is that unlike on gold which has an ordered atomic surface which promotes a dense and ordered monolayer [17], such monolayers formed on oxides and carbon materials are generally much more heterogeneous and porous. Additionally, some of these materials, such as indium tin oxide, can become unstable under electrochemical interrogation and dissolve into solution over time [16].

We hypothesize that an ultra-thin oxide layer may serve as an alternative blocking layer because such layers are known to rapidly hydrate with water and are electrochemically porous as shown in electrowetting devices [18]. A further speculated advantage of such ultra-thin oxide blocking layers is that the function of the blocking layer can be separated into two separate material layers. The first layer is the inorganic oxide or nitride layer which is electrochemically porous enough to allow robust redox electron transfer (Fig. 1 right) but dense enough to block oxygen reduction currents. The second layer is then a covalently bonded monolayer chemistry which supports robust antifouling properties. Additionally, such thin oxides are highly manufacturable as a critical material in the global semiconductor industry [19, 20].

Presented here is an evaluation of ultra-thin inorganic oxide and nitride films as an alternative to self-assembled blocking monolayers. Silicon oxide, silicon nitride, and aluminum oxide films were deposited by electron beam evaporation (EB) or atomic layer deposition (ALD) at thicknesses of several nanometers to mimic the required electrical capacitance of a conventional monolayer blocking layer (3.40 – 4.72 μF/cm^2^) [21]. These oxide films were fundamentally characterized for stability over a 7-day period and provided poor protection against interfering redox currents from dissolved ferricyanide (~ 150 – 300 μA/cm^2^) and from oxygen reduction interference (~ 30 – 60 μA/cm^2^). The oxide films were then integrated into an EAB sensor format using the well-known aptamer for phenylalanine, showing lowered binding affinity and weak sensor response with ~20 % change in methylene blue redox current compared to the expected 50 % previously published on gold [22]. Results further show that if higher porosity oxide or nitride films are used, they may be more stable over multiple days but have significantly higher initial interferent currents than denser films. It is speculated that these oxide and nitride films dissolve over several days in an aqueous environment. While oxide and nitride films fail to improve upon the performance of thiol-blocking layers on gold electrodes, they may provide utility in unexplored applications beyond biosensors by allowing alternate electrode materials to be used. Therefore, this work can be used to provide alternative blocking layers for biosensing to thiol-based monolayers on gold. Lastly, our research group strongly values the importance of publishing work that did not meet initial hypotheses, in the interest of answering questions that haven’t been considered, thus saving other researchers time and effort in their pursuits [23].

### 1.1. Rationale for SiO_2_

SiO_2_ was chosen initially as the preferred oxide or nitride blocking layer for several reasons. In theory, SiO_2_ has properties that can create an acceptable blocking layer with greater stability and versatility compared to blocking groups based on gold-thiol chemistries. Firstly, SiO_2_ can be deposited with a wide range of porosities that rapidly hydrate with water, which in theory could allow redox electron transfer while reducing the diffusion of interferents such as oxygen to the electrode surface [24]. Secondly, SiO_2_ can be easily modified with a wide range of antifouling chemistries [25]. Thirdly, SiO_2_ has a low dielectric constant of ***ε***_***r***_ ~ 4 which reduces capacitive background currents at higher voltammetry scan frequencies and rates. Fourthly, SiO_2_ can be deposited on nearly any electrode material at low temperatures and at higher temperatures can even be deposited on rough or porous electrodes using conformal deposition methods such as ALD.

A conventional self-assembled alkylthiolate monolayer is typically 1 nm thick or less and has a dielectric constant of ***ε***_***r***_ ~ 2–3. A SiO_2_ layer has approximately double the dielectric constant of an alkylthiolate monolayer ***ε***_***r***_ ~ 4. According to a simple capacitance calculation of ***C*** = ***ε***_***r***_***ε***_0_ */****d*** a SiO_2_ blocking layer should be approximately 2 nm thick or greater so that conventional square-wave frequencies can be utilized (10’s to 100’s of Hz, ***f***_***c***_ = 1*/*(2***πRC***)). A more rigorous calculation of cutoff frequency ***f***_***c***_ is also provided in appendix A1 and confirms that the SiO_2_ should be > 1.6 nm for ***f***_***c***_ > 1000 ***Hz***. This of course assumes alkylthiolate and SiO_2_ blocking layers have low porosity (< 10 %) such that they further have low levels of hydration by water in blocking layers, because water could significantly raise the blocking layer dielectric constant (***ε***_***r***_ ~ 80 for water) [26].

## 2. Materials and methods

### 2.1. Materials

4” 100 nm gold-coated P-type wafers, 30 % hydrogen peroxide, ≥ 99.9 % methanol, nuclease free molecular biology water, 1x phosphate buffered saline (PBS), ≥ 99.9 % dimethyl sulfoxide (DMSO), tris(2-carboxyethyl) phosphine (TCEP), ACS grade potassium hexacyanoferrate, 99 % 6-mercapto-1-hexanol (MCH), and ≥ 98 % L-phenylalanine were purchased from Sigma-Aldrich (St. Louis, MO, USA). 0.001 – 0.005 Ω cm, 500 μm, P-type, single side polished wafers were purchased from Alpha Nanotech (Vancouver, CA). Copper tape (Kraftex), Kapton polyimide tape ½ ” 2 mil thick (Bertech), hot glue (kejector), 1 ” water soluble tape (SmartSolve), and 25L controlled temperature enclosures (Happybuy) were purchased from Amazon (Seattle, WA, USA). ACS grade sulfuric acid, 99 %, 3-Maleimidopro-pionic acid N-hydroxysuccinimide ester, and ACS grade sodium chloride were purchased from Fisher Scientific (Hampton, NH, USA). Pure methylene blue and 99+ % potassium ferricyanide were purchased from ACROS Organics, now part of Fisher Scientific (Hampton, NH, USA). Ag/AgCl reference (CHI111), and Pt counter electrodes (CHI115) were all purchased from CH Instruments (Austin, TX, USA). Phenylalanine aptamer [/CG ACC GCG TTT CCC AAG AAA GCA AGT ATT GGT TGG TCG/] with conjugated methylene blue and thiol ends, was purchased from Integrated DNA Technologies (Coralville, IA, USA). Phenylalanine was chosen as an ideal aptamer because it is well established in literature for electrochemical measurements. Amino propyl dimethyl ethoxy silane (APDMES) was purchased from Gelest (Morrisville, PA). All electrochemical measurements were taken with a CHI650E potentiostat and CHI684 multiplexer from CH Instruments. (Austin, TX, USA).

### 2.2. Substrate preparation

Sensors were fabricated on gold-coated silicon substrates which were cut into segments approximately 1 cm wide and 1.5 cm long using a diamond scribe. All substrates were then cleaned using piranha solution (1: 3 H_2_O_2_: H_2_SO_4_) for 20 min and thoroughly rinsed with water then ethanol. The substrates were then baked at 65 °C for 30 min to remove any remaining ethanol and allowed to cool to room temperature while covered for 30 min. Substrates were then attached to a glass microscope slide using double sided tape and water-soluble tape for coating with oxide and nitride films.

### 2.3. Oxide and nitride coatings

SiO_2_ coated electrodes were made using two techniques, EB and ALD. ALD samples were prepared by Okyay Tech (Palo Alto, CA, USA) to the desired thickness. EB SiO_2_ samples were prepared using a Temescal FC-1800 with a CV-12 power supply. Deposition was carried out at an accelerating voltage of 9 kV DC with a filament current of 26 A and an emission current of 10 mA. Si_3_N_4_ coated electrodes were made using a Denton Discovery 24 with an Advanced Energy Cesar RF power supply. Deposition was carried out at 150 W RF with argon and nitrogen gas at 2 sccm and 1 sccm respectively at 0.51 Pa of pressure. A bias voltage of 210 V was used. Al_2_O_3_ deposition was also done with the Denton Discovery 24 but at 125 W RF with argon gas at 5 sccm at 0.72 Pa. A bias voltage of 32 V was used. Deposition for all these films was carried out to the desired thicknesses based on previous calibration of the deposition system for much thicker oxide films. After coating, substrates were removed from the glass slide by briefly soaking in water and immediately dried under N_2_, piranha cleaned using the above stated process a second time, dried, and then kept covered until silane functionalization.

### 2.4. Functionalization into electrochemical working electrodes

**Fig. 2.**
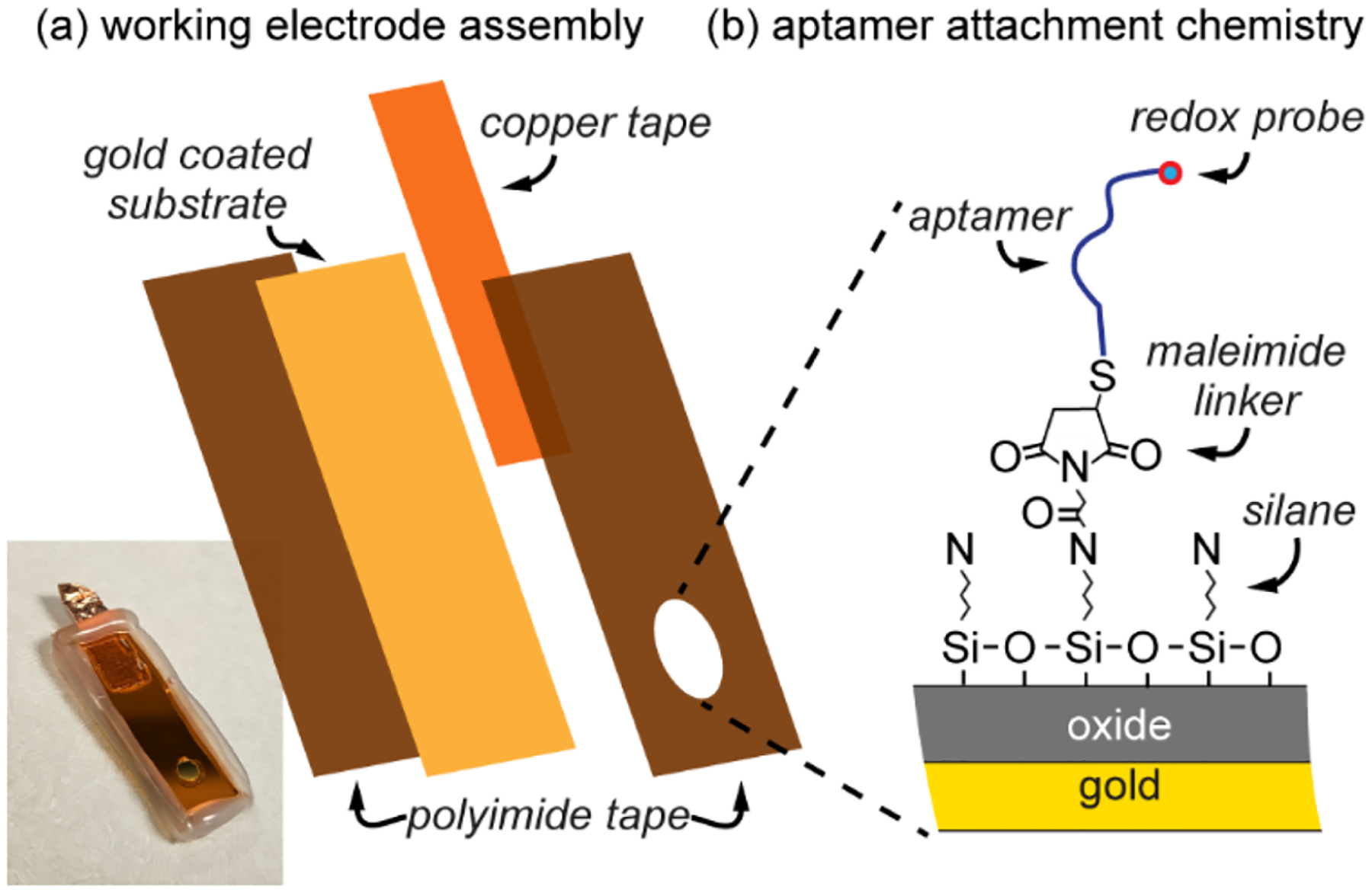
Scheme of working electrode physical and chemical assembly. (a) Physical layers of working electrode assembly sealed with polyimide tape and hot glue (shown in photo insert) built on 1.5 by 1 cm wafer chips. (b) chemical modification of working electrodes for aptamer attachment to silane functionalized oxide.

To coat chips with aptamer, APDMES was used to bridge the oxide and nitride layers to the thiolated aptamer via a maleimide linker. Silane coatings were applied via methanol solution phase deposition. Oxide coated chips were submerged in a stirred methanol bath with 1 % V/V APDMES. Chips were submerged for 18 h under constant stirring before being removed from the methanol bath. Chips were then rinsed with methanol and dried under N_2_. Once dry, chips were baked at 120 °C for 20 min and then allowed to cool to room temperature. Once cool, adhesive copper tape was connected to the gold electrode side of the substrates and then electrically insulated with adhesive polyimide tape exposing a 3 mm diameter hole to restrict the active site for electrochemical measurement to an area of 7.07 mm^2^. The back of the substrate was also covered with polyimide tape and the two layers of polyimide tape were sealed with hot glue to prevent water penetration (Fig. 2a).

### 2.5. Aptamer preparation and deposition

Aptamers were resuspended to 100μM from a lyophilized pellet using molecular biology H_2_O and kept at −20 °C until use. For use on electrodes, equal parts of the resuspended aptamer solution and 0.5 M TCEP in molecular biology H_2_O were mixed and allowed to rest for 1 h in a dark box to ensure complete reduction of disulfide bonds. After resting, the aptamer was diluted to 4 μM using 1x PBS with 5 M NaCl. Aptamer concentration was confirmed via absorbance at 260 nm using a Nano-drop UV/Vis spectrophotometer. This solution was then diluted to a working concentration of 500 nM using 1x PBS with 5 M NaCl.

To functionalize the oxide films with phenylalanine aptamer, the following steps were taken. First, a maleimide ester was used to bind free hydroxyl groups on the silane functionalized dielectric surface to the thiol terminated aptamer. Maleimide solution was made to 1mM concentration by first suspending 1.3 μg of maleimide in 500 μL of DMSO. Once the maleimide was fully dissolved, 4 mL of molecular biology H_2_O was added then 500 μL of 10x PBS was added. The solution was mixed well and 30 μL was deposited onto the active site of electrodes and allowed to rest for 30 min in a humid dark box. After maleimide functionalization, electrodes were rinsed with deionized (DI) water and dried under a flow of N_2_ gas. Once dry, 25 μL of working aptamer solution was then deposited onto the active sites of electrodes and allowed to rest in a humid dark box for 12 h for functionalization. The final working electrode assembly is shown in Fig. 2b.

### 2.6. Longevity testing

To test how the oxide and nitride blocking layers would perform over time in an *in-vitro* environment, several electrochemical tests were employed. Electrodes were placed into 1x PBS at 33 °C, an average temperature equivalent to that experienced by wearable biosensors like glucose monitors [27]. An initial scan was immediately performed using cyclic voltammetry (CV) in the voltage range of methylene blue, −0.05 to −0.50 V. Immediately after an initial 1x PBS CV scan, the working electrodes were placed into 1mM ferricyanide solution, and a CV scan was taken between 0.7 and −0.4 volts to confirm redox transfer from ferricyanide.

After all initial CV scans were taken, working electrodes were rinsed with DI H_2_O and placed back into 1x PBS. During longevity testing, the working electrodes were continuously scanned every 5 min using square wave voltammetry from −0.05 to −0.50 V. Each day of testing, a CV scan was performed in 1x PBS. After this CV, electrodes were continuously scanned every 5 min. Every other day, electrodes were removed from 1x PBS and placed into 1mM ferricyanide and a CV was taken between 0.7 and −0.4 V. After ferricyanide testing, electrodes were rinsed with DI H_2_O and placed back into 1x PBS to be scanned continuously every 5 min. These longevity tests were performed for 1 week at 33 °C. 1x PBS solutions were replaced every 3 days to mitigate any interfering effects of evaporation.

### 2.7. Aptamer testing

Aptamer functionalized working electrodes were only constructed on SiO_2_ coated working electrodes. For phenylalanine titration testing, specifically 8 nm of EB deposited SiO_2_ was used. Functionalized working electrodes were rinsed of any remaining aptamer solution and dried under N_2_ stream and then immediately placed into a 1x PBS beaker at room temperature. An initial CV scan of all working electrodes was taken between −0.05 and −0.5 V and then the sensors were stabilized by repeated square wave voltammetry measurements until sensors had no discernible difference in peak height between successive scans. Stabilization scans were performed to remove any remaining loosely bound aptamer that may have stuck to the oxide surface, the amine termination of the silane or any exposed gold. After stabilizing the working electrodes, they were scanned using square wave voltammetry at 5, 10, 20, 30, 60, 100, 120, 150, 180, 200, 250, 300, 400, 500, 750, and 1000 Hz. After observing the most promising voltammograms at 120, 300, and 500 Hz, the working electrodes were repeatedly scanned at 120, 300, or 500 Hz until there was no discernible difference in peak height between successive scans. After sensors were stabilized, the working electrodes were titrated with phenylalanine for the test frequencies of 120, 300, and 500 Hz. After each titration of phenylalanine was added to the bulk solution, the solution was mixed for 60 s before the next set of voltammograms were acquired. After the titration was completed, the working electrodes were rinsed with DI H2O and placed into a blank solution of 1x PBS for regeneration.

### 2.8. Data processing

All data was processed using MathWorks MATLAB and Microsoft Excel and then plotted using Excel.

## 3. Results and discussion

### 3.1. Demonstration of an aptamer sensor on an oxide blocking layer

**Fig. 3.**
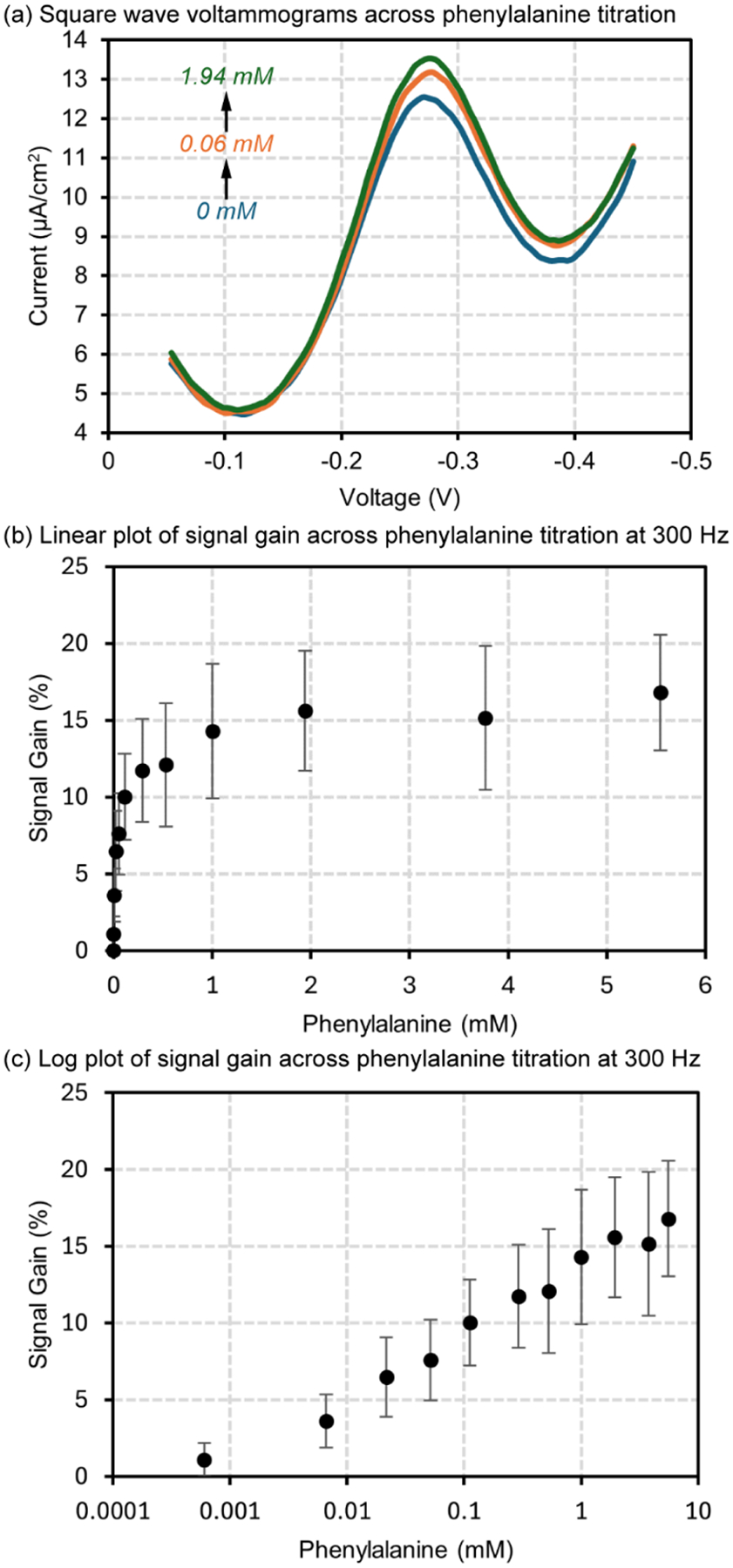
Phenylalanine titration using 8 nm SiO_2_ blocking layer on gold. Phenylalanine aptamer is attached via a maleimide linker to the SiO_2_. These sensor results are for n = 3 electrodes and are only for freshly prepared and tested sensors. These measurements were taken using square wave voltammetry at an amplitude of 50 mV, and a frequency of 300 Hz. (a) square wave voltammograms at low, medium, and high concentrations. (b) linear titration curve for phenylalanine. (c) log titration curve for phenylalanine.

**Fig. A2.**
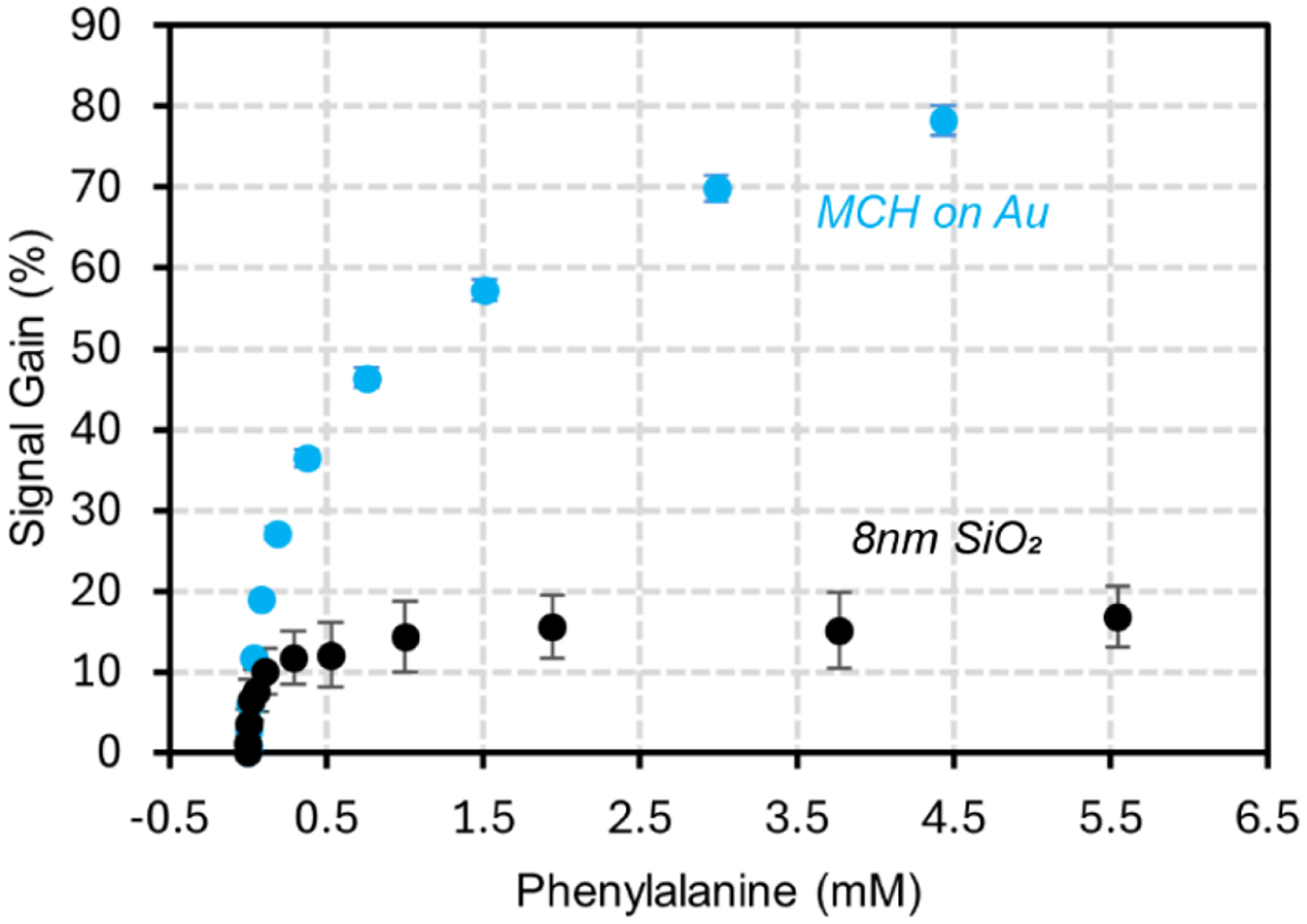
Signal gain plotted over concentration for electrodes prepared using conventional methods on gold with a MCH and using 8 nm oxide as the blocking layer. Oxide blocking layers are outperformed by conventional methods and have a >60 % drop in maximum achievable signal gain.

Fig. 3 shows the raw square wave voltammograms for a phenylalanine aptamer sensor fabricated on 8 nm SiO_2_. The phenylalanine aptamer sensor with an oxide blocking layer exhibits a range of detection and binding affinity lower than phenylalanine aptamer sensors prepared with alkylthiolate blocking layers. The calculated binding constants were 12 μM and 400 μM for SiO_2_ and alkylthiolate, respectively. Additionally, the maximum sensor response is ~ 15 – 20 % and lower than the ~ 50 % response observed for phenylalanine aptamer sensors with alkylthiolate blocking layers in literature [22] (Fig. 3 b and c). Included in the appendix A3 is a comparison between electrodes prepared on oxide vs on gold showing the differences in maximum achievable signal gain (Appendix Fig. A2). The origins for this reduced response is the result of decreased packing density driven by inefficient binding between the silane, maleimide, and thiolated aptamer as well as interactions between the inorganics and phenylalanine. Packing densities for SiO_2_ sensors are 0.768 × 10^12^ aptamers/cm^2^ while sensors prepared on gold have a packing density of 5.97 × 10^12^ aptamers/cm^2^. This ~ 8x difference in packing density could contribute to the lower saturation concentration and sensitivity observed in the SiO_2_ sensors. Additionally, phenylalanine is hydrophobic and could be sticking to the oxide surface preventing proper binding with the aptamer. This nonspecific binding could lower sensitivity by hindering aptamer folding and not binding to aptamers. Typical working electrodes with alkylthiolate blocking layers can have redox (baseline adjusted) currents of ~ 8 μA/cm^2^ and the peak current for the voltammograms in Fig. 3 are similar at ~ 8 – 9 μA/cm^2^ [12]. While the voltammograms do show evidence of oxygen reduction at potentials more negative than the methylene blue redox peak, the oxygen reduction current is not dominantly large during these initial titration tests. However, as will be seen in the next section on longevity, the oxygen reduction current can exceed ~ 100 μA/cm^2^ after prolonged continuous operation.

### 3.2. 7-Day longevity testing of oxide and nitride blocking layers

Evaluation of blocking layer longevity was done through two different measurements to evaluate degradation and changes in the dielectric surface including: (1) ferricyanide redox current peak height and (2) oxygen reduction current at −0.45 V.

**Fig. 4.**
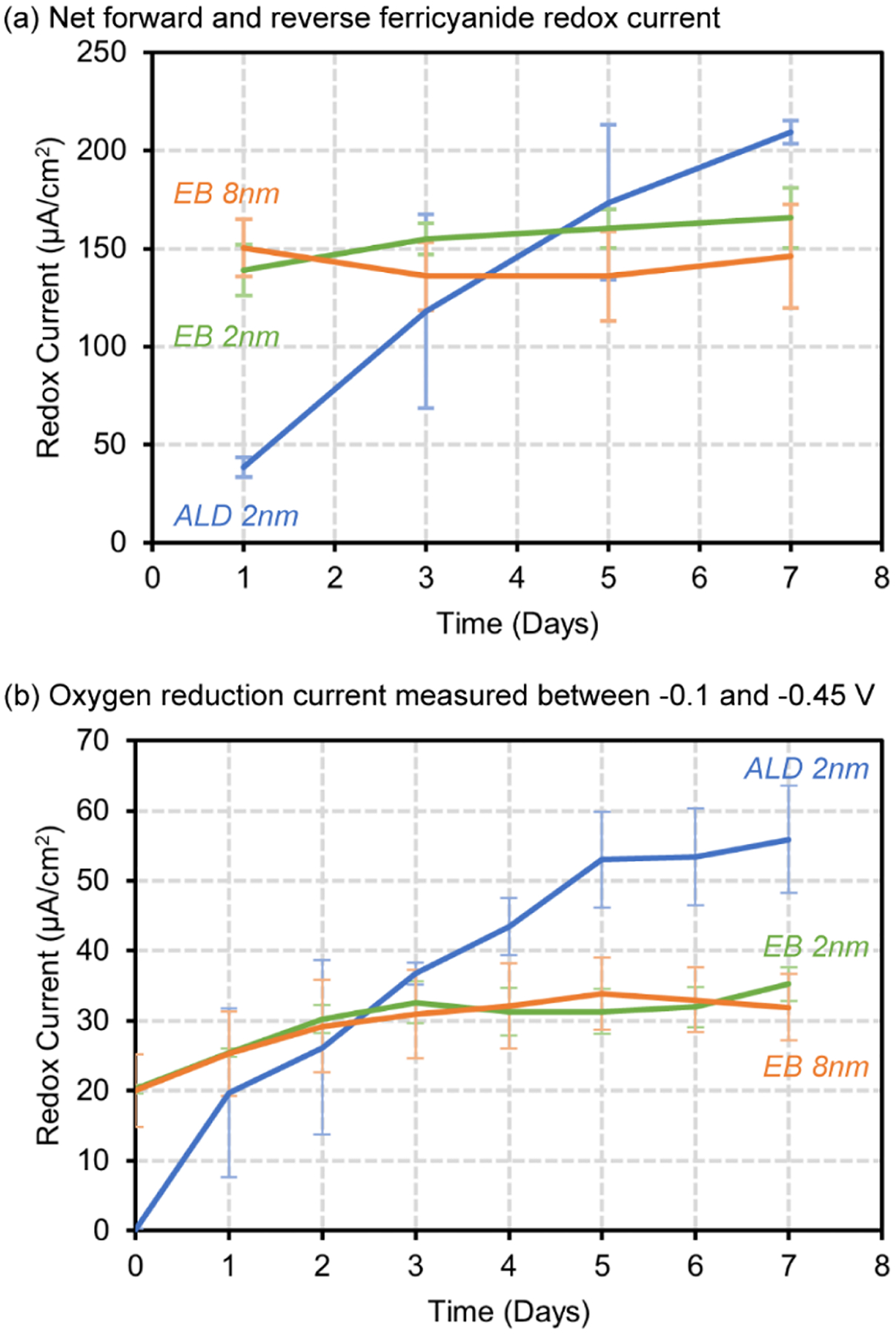
Longevity experiments of SiO_2_ deposited by ALD and EB to evaluate long-term stability of oxide blocking layers. (a) Interfering current from a solution phase redox reporter, ferricyanide. (b) Oxygen reduction current measured indirectly using the difference in current between −0.45 V and −0.1 V using CV.

The ferricyanide redox current was used to capture non-specific redox currents because ferricyanide relies on proximal reactions with gold to exchange electrons through cyanide bridges [28]. Because of this, ferricyanide would need to directly contact exposed gold to exchange electrons, allowing us to indirectly track exposed gold over time [29]. This reaction will gradually etch the gold over time but is insignificant compared to other degradation mechanisms discussed later. By tracking ferricyanide forward and reverse current peaks over time, the total redox current could be used as a proxy for blocking layer breakdown. While this is not a direct measurement of film breakdown over time, it will still provide insights into the degradation of the inorganic materials. As shown in Fig. 4a, the EB samples ferricyanide currents do not significantly change over time while the ALD samples change significantly. Both EB samples maintained redox currents around 150 – 180 μA/cm^2^ over 1 week while the ALD samples changed from 50 μA/cm^2^ to 200 μA/cm^2^. It is important to note the difference between initial ferricyanide current between the EB and ALD samples (150 μA/cm^2^ vs 40 μA/cm^2^, respectively).

Further longevity analysis was performed using changes in oxygen reduction by CV and measuring the difference in current between −0.45 and −0.1 V of the forward reduction scan instead of analyzing oxygen reduction peaks beyond −0.5 V. If full oxygen spectra were to be measured, it would have been far beyond the scanning potential range used for methylene blue tagged sensors and would not be representative of real-world device usage. Fig. 4b reveals that oxygen reduction progressively increases for all samples with the biggest change observed in the ALD samples, 1 μA/cm^2^ to 55 μA/cm^2^, while the EB samples had a lesser but still significant increase in oxygen reduction over time, 20 μA/cm^2^ to 35 μA/cm^2^ and 20 μA/cm^2^ to 31 μA/cm^2^ for 2 and 8 nm EB samples, respectively (Fig. 4b).

The initial differences between the ALD samples and the EB samples can be attributed to the natural porosity created by the two deposition techniques. EB creates inherently more porous layers than uniform conformal layers achieved with ALD [24]. Having a higher porosity, it is natural that the EB samples would start with a higher redox current as the ferricyanide could more easily diffuse through the dielectric to the gold surface to exchange electrons. This porosity argument is also supported by oxygen reduction measurements. It is speculated that the differences between the two deposition types produced differences in porosity and difference in pore stability: larger, more stable pores in the EB samples and smaller less stable pores in ALD samples.

Ultimately, SiO_2_ was found to work as a suitable replacement for thiol blocking layers for real time measurements but could not reach lifetimes of multiple days or weeks using the deposition methods tested.

### 3.3. 7-Day longevity testing of silicon nitride and alumina blocking layers

**Fig. 5.**
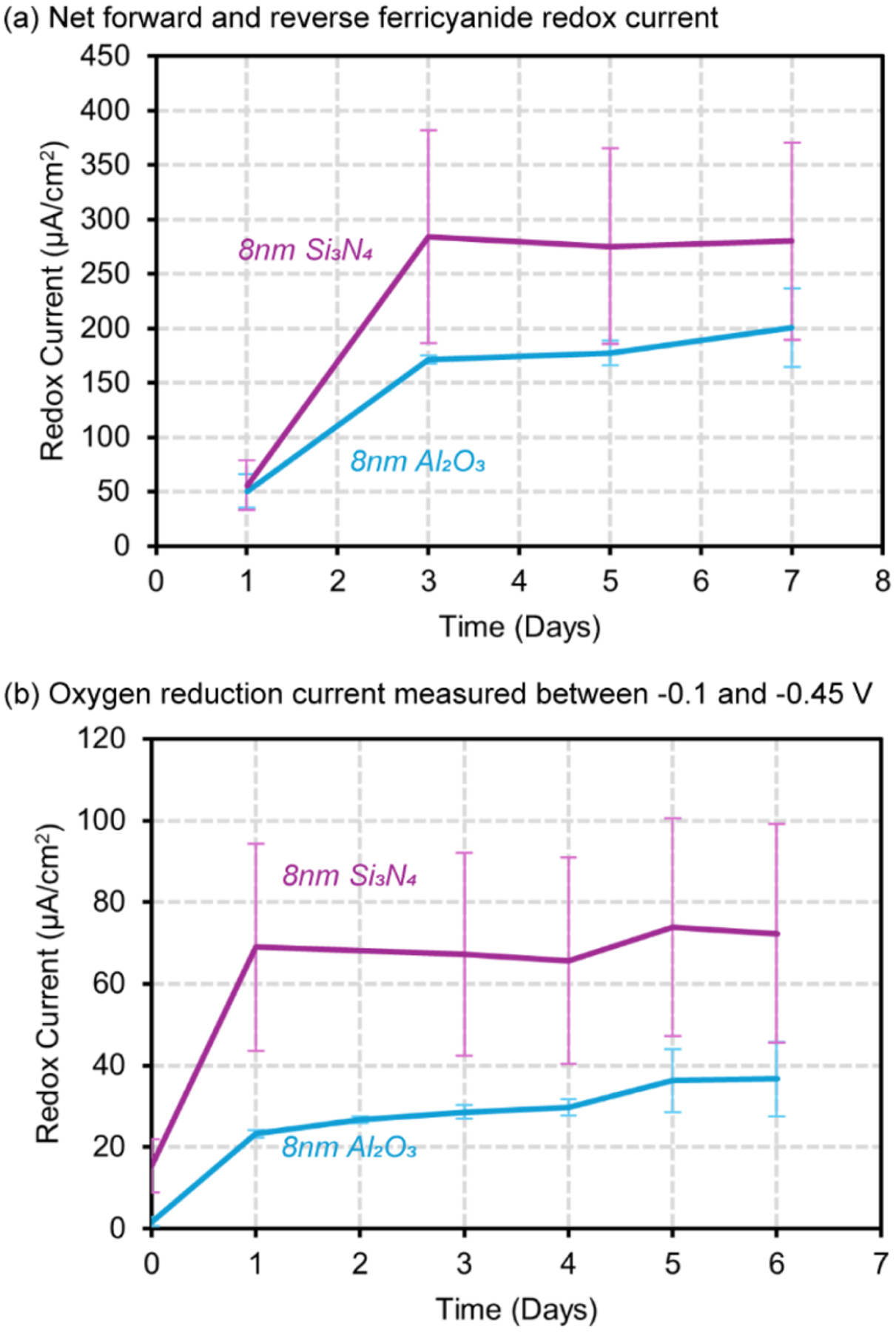
Longevity experiments to evaluate long-term stability of alternative dielectric blocking layers deposited by sputtering: Si_3_N_4_ and Al_2_O_3_. These materials show significant changes in measured currents across all measured variables with Si_3_N_4_ having the highest amount of change. (a) Interfering current from a solution phase redox reporter, ferricyanide. (b) Oxygen reduction current measured indirectly using the difference in current between −0.45 and −0.1 V using CV.

Because of the inadequacies observed in SiO_2_, alternative dielectric materials that were not within the initial scope of materials were explored. Al_2_O_3_ and Si_3_N_4_ were both considered as alternatives to SiO_2_ as they have lower dielectric constants compared to many other dielectrics and maintain the high level of film customizability like that achievable for SiO_2_ [30,31]. These materials were evaluated similarly to the SiO_2_ including ferricyanide interferent redox current, and oxygen reduction current. These alternative dielectrics exhibit more drastic changes between starting and final currents compared to the SiO_2_ samples. Over time, both nonspecific redox currents from ferricyanide and oxygen reduction increased significantly and therefore neither of these alternate oxide or nitride films were adequately stable for multi-day use (Fig. 5).

### 3.4. Discussion

As seen above, the tested oxide and nitride films are not suitable blocking layers for long-term operation of electrochemical biosensors. They fail in blocking nonspecific redox currents from interferent currents like ferricyanide and oxygen reduction. While *in-vivo* dissolved oxygen concentrations are lower than atmospherically available oxygen, any variation in dissolved oxygen *in-vivo* will affect sensor reliability by interfering with the redox reporter current from the aptamer probes [32]. Literature supports that the degradation observed in oxide and nitride blocking layers is due to dissolution even near neutral pH levels by hydrolysis (Table 1). Constant exposure to water and the higher temperature of the human body would exacerbate thin film degradation making them ineffective for long term sensing applications.

**Fig. A3.**
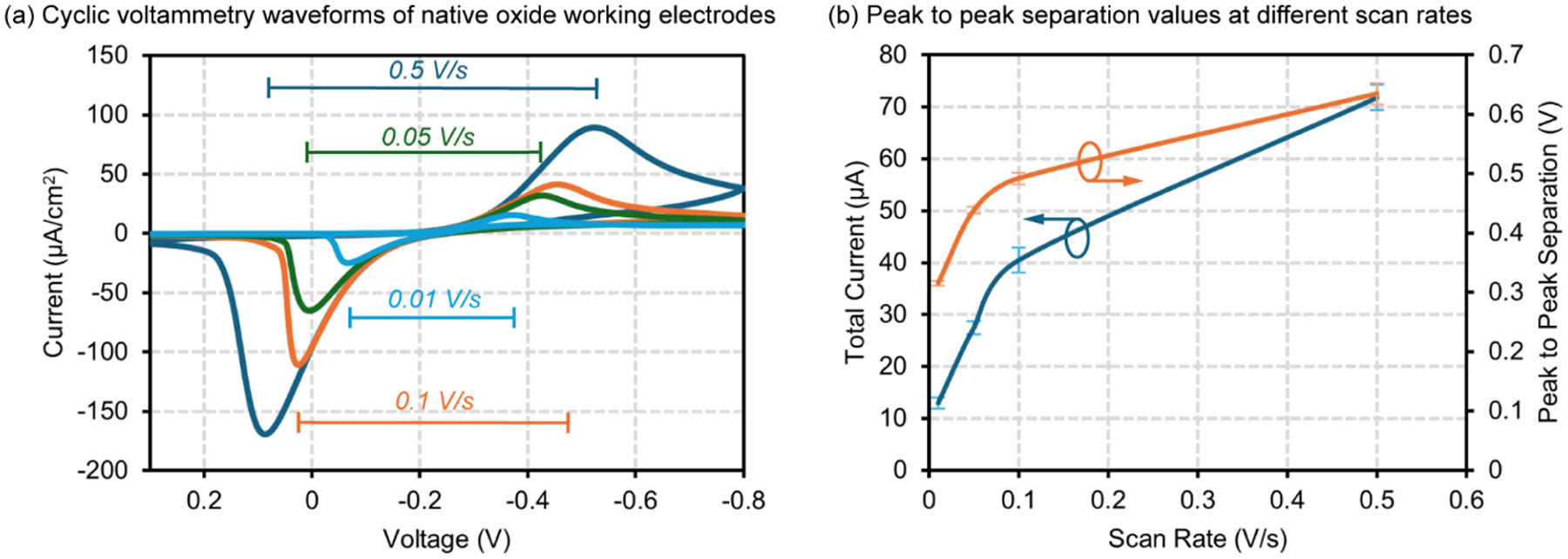
Low resistivity wafers with native oxide used as the working electrode in a three-electrode cell. (a) Waveforms produced from scanning in methylene blue indicating irreversibility but sufficiently high current density. (b) Peak to Peak voltage separation and total redox current plotted against scan rate demonstrating scan rate dependence of scanning indicative of redox transfer and not interfering processes.

**Fig. A4.**
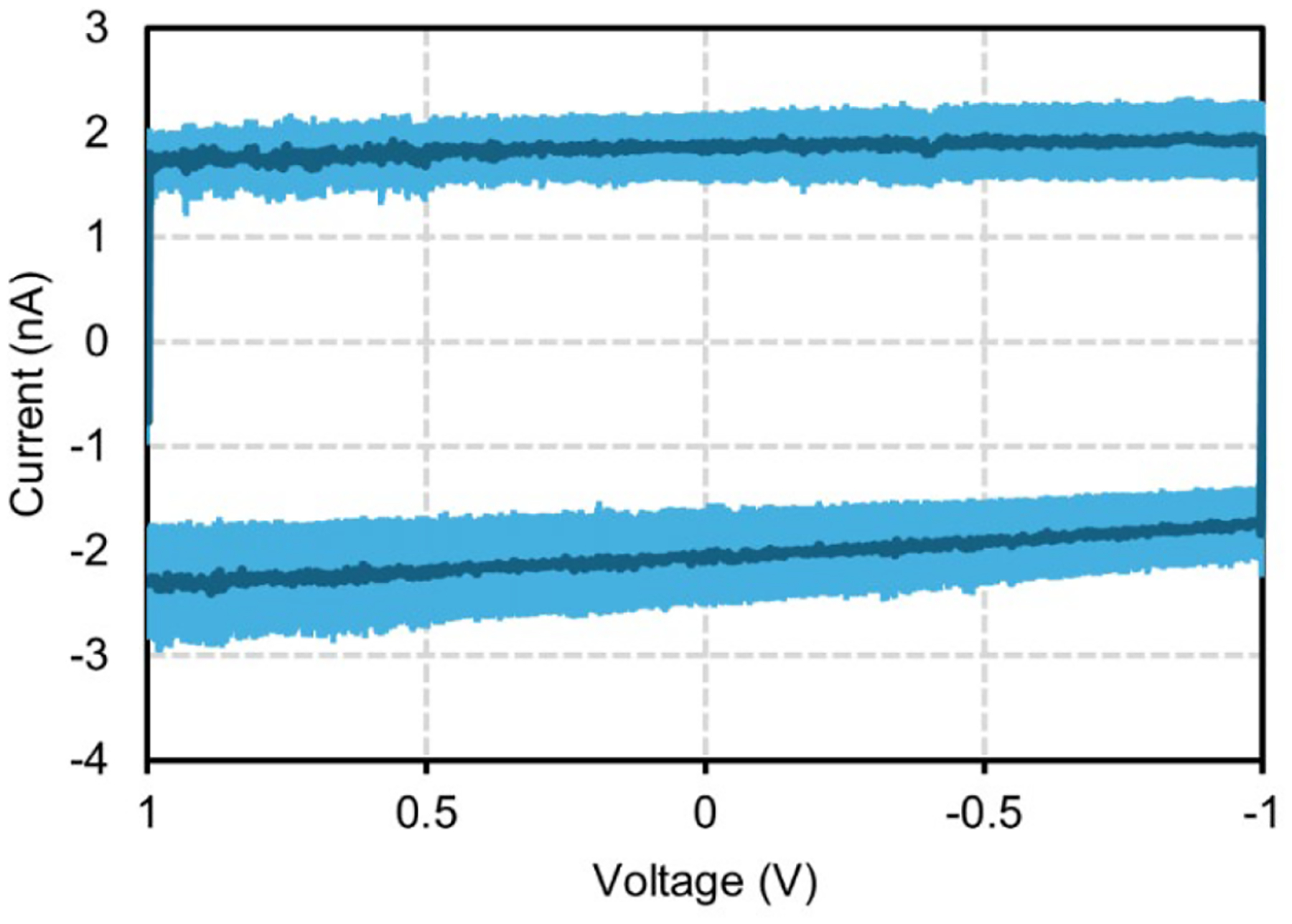
CV waveforms produced by 4nm thermal oxide working electrodes prepared on low resistivity silicon electrodes. No apparent peaks were presented, and no confounding electrochemical processes were observed, like oxygen reduction.

An alternative to the thin films tested here might be thermally grown or plasma-enhanced chemical vapor deposited (PECVD) oxide (Table 1), or SiC films, all which show slower dissolution rates than sputtered films as corroborated by mass spectrometry, and atomic force microscopy [33, 34,36]. Due to their potential for increased stability, thermally grown SiO_2_ films were also evaluated on low resistivity Si wafers via electron transfer from a solution phase redox reporter (methylene blue). Native and thermal oxide films < 4 nm thick could pass redox current from the solution phase redox reporter but, due to the increased SiO_2_ film density, the redox system was irreversible as evidenced by the large peak to peak separation in CV. (appendix Fig. A3) Additionally, thicker thermal SiO_2_ films (4 nm) were evaluated and could not pass any observable methylene blue current (appendix Fig. A4). Because of this, thermally grown films, while more stable, were inadequate for biosensor applications. PECVD films were not explored and are less desirable due to the high temperatures and complexity required to deposit them.

It appears, based on this work, that oxide films are too unstable if they are to also be adequately dense for limiting interferent redox electron transfer. Conversely, if they are adequately stable such films might be too passive as electrochemical barriers for interferent electron transfer. This leaves SiC or TiO_2_ as unexplored and potential replacement blocking layer materials as they show excellent stability in low oxygen aqueous environments like those observed in the body [32,36,37]. However SiC and TiO_2_ have higher dielectric constants of ~ 10 and ~ 24 – 57, respectively, which is not ideal as it may cause capacitive charging currents to dominate over redox currents when using analytical techniques such as square wave voltammetry [38]. Perhaps lower dielectric constant (~2) fluorinated insulators (PECVD deposited fluo-rocarbons) would provide a stable and lower dielectric constant alternative. Unfortunately, such materials were not available for this study, and such materials may bring their own challenges as the more inert the material the more challenging it is to covalently link sensing elements such as aptamers to the surface.

While the evaluated oxide and nitride films evaluated here do not show value as a blocking layer for working electrodes in a biosensor, there may be speculative value as a protective layer for a reference/counter electrode. On the reference or counter electrode, thicker yet still porous films would be porous enough to allow for electron transfer, not impeding their function, but could also dissolve at a controlled rate removing fouling proteins that can cause drift [7]. By removing the fouling proteins at a controlled rate, it might be possible to keep the reference and counter electrodes clean making implantation into the body simpler by reducing the complexity of the implanted electrodes. Such self-cleaning reference and counter electrodes would also make it easier to use software correction of drift by having more gradual potential drift patterns and making it easier to follow the redox tag peak current as it shifts [10].

## 4. Conclusions and perspective

To increase lifetimes of EAB biosensors, oxide and nitride films were explored as an alternative blocking layer material to reach the 3-day and 2-week goals set by commercially available bio monitors. While the oxide and nitride films worked as a blocking layer for aptamer biosensors, they ultimately failed to meet the benchmarks required due to hydrolysis degradation of the films. Because of this, these films should largely be avoided for blocking layers on working electrodes but might have benefit on reference and counter electrodes without biorecognition elements allowing them to self-clean over time.

This study has further generated our own reflections on how to move past thiol-based monolayers in general. In the introduction, we discussed an ideal consideration of blocking layer density. Blocking layer density is critical to suppress diffusion of common redox interferents to the electrode surface. Furthermore, poor blocking layer density or high blocking layer defect density can be challenging by opening up locations for surface fouling interactions. We therefore speculate that gold surfaces are quite unique because of the ordered crystal planes which provide a tightly spaced and defect free domains for blocking layer assembly. In fact, the crystal planes on rough gold are just about perfect size to promote low-defect densities limited primarily to gold step edges where alkythiolate stability is strong due to small defect size [14]. Therefore, when we speculate on ideal alternate systems to be explored in the future, we think of systems such as graphene which can potentially promote more tightly ordered monolayers. In addition, semi-ordered (polycrystalline) dielectrics of nitrides or fluorides may have potential value if they can provide low domain order and unlike oxides a low dissolution rate in aqueous solutions.

Based on the results here and recent advances in thiol longevity, we conclude that blocking layers for biosensors should rely on further improvements to thiol-on-gold monolayers to improve stability and foulant resistance as thiol monolayers have been further established as reliable for long term scanning [12]. These monolayers have proven themselves in terms of stability *in-vivo* and further work should focus on finding the limits of their useful lifetimes and how to incorporate antifouling chemistry within the thiol monolayer to broaden the scope of biosensors which can be translated into real world devices.

## Figures and Tables

**Table 1:** Degradation rates for silicon oxide and nitride thin films at room temperature and 37 °C. The mechanism for hydrolysis of silicon oxides is SiO_2_ + 2H_2_O ↔ Si (OH)_4_ Silicon nitride hydrolyzes in aqueous solution in two steps: (1) oxidation into silicon oxide (Si_3_N_4_ + 6H_2_O ↔ 3SiO_2_ + 4NH_3_) and (2) hydrolysis of silicon oxide (SiO_2_ + 2H_2_O ↔ Si(OH)_4_), where the overall reaction is Si_3_N_4_ + 12H_2_O ↔ 3Si(OH)_4_ + 4NH_3_ [33,34]. For Al_2_O_3_ films hydrolysis happens by hydrolysis of Al-O bonds $\left({\text{Al}}_{\text{2}}{\text{O}}_{3}+{\text{H}}_{2}\text{O}↔2\text{Al}{\left(\text{OH}\right)}_{4}^{-}\right)$ [35].

All for pH=7.4	Room temp	37 °C
SiO_2_ EB	~5 nm/day	~10 nm/day
SiO_2_ PECVD	~0.05 nm/day	~0.1 nm/day
SiO_2_ thermal	~0.0005 nm/day	~0.003 nm/day
Si_3_N_4_ PECVD	~0.07 nm/day	~0.3 nm/day

**Fig. A1.**
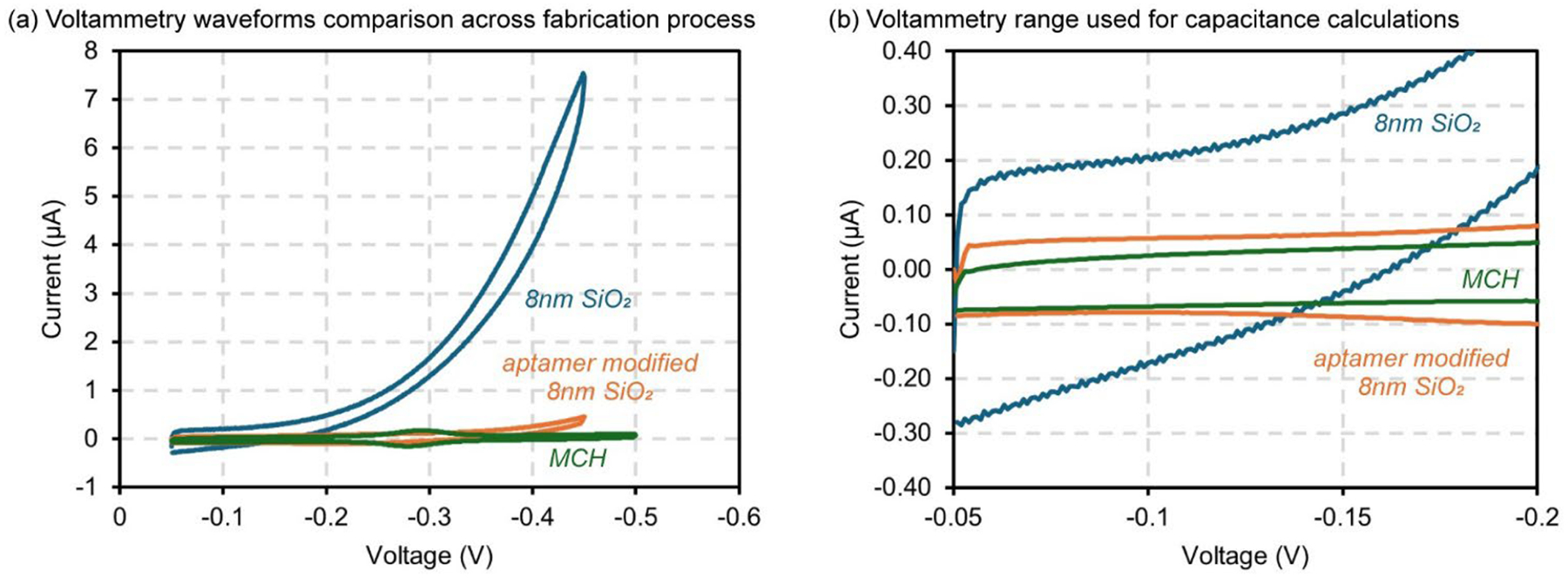
(a) Representative voltammograms of inorganic sensors at different stages of the fabrication process including the 8 nm SiO_2_ with and without aptamer modification. (b) Voltage range used for capacitance measurements and calculations. 8 nm SiO_2_ without aptamer modification has the highest capacitance while 8 nm SiO_2_ with aptamer modification exhibits a similar capacitance to thiol based monolayers, specifically MCH.

## Data Availability

Data will be made available on request.

## Appendix

### A1. Calculations for silicon dioxide thickness

For this calculation, we will assume that we have an infinitely large area of a planar electrode to treat transmission lines as perfectly perpendicular to the electrode surface. We will also assume we have pure, nonporous SiO_2_, and we are working in undiluted blood. This allows us to treat the circuit between the counter and working electrodes as an RC circuit which behaves as a low pass filter and calculate cutoff frequencies based on thickness. Calculations will be based off commercially available continuous glucose monitors which have an electrode depth, *L*, of 5 mm into the body [39]. Using this needle depth would be the worst-case scenario for working and counter electrode separation. By using the parallel plate capacitor, resistivity, and RC circuit equations we can rearrange to solve dielectric thickness, *d*, as a function of cutoff frequency (Eqs. (A1)–(A4)). By solving Eq. (A4) using the resistivity of blood 100 Ωcm, *ρ* [40], permittivity of free space, *ε*_*0*_, dielectric constant of 3.9, *ε*_*r*_, [41] and a cutoff frequency, *f*_*c*_, of 1,000 Hz, we get that our SiO_2_ blocking layer must be ≥ 1.1 microns to pass 1,000 Hz. Knowing this we can inform the minimum height of SiO_2_ needed for subsequent testing with aptamer probes.

(A1)
$$C=\frac{\varepsilon_{r}\varepsilon_{0}A}{d}$$

(A2)
$$\tau =RC=\frac{1}{2\pi f_{c}}$$

(A3)
$$R=\frac{\rho L}{A}$$

(A4)
$$d=f_{c}*(2\pi (\rho L)(\varepsilon_{r}\varepsilon_{0}))$$

### A2. Comparison of capacitance between inorganic and aptamer modified surfaces

To understand the sensor’s operational mechanism, we measured capacitance at various stages of fabrication. This analysis helps us assess the quality of our deposition techniques and how they influence the observed electrochemical performance. Capacitance was determined from cyclic voltammetry (CV) scans by calculating the average current at −0.150 V in phosphate-buffered saline (PBS) at a scan rate of 100 mV/s. Specifically, we calculated the difference between the forward and reverse currents, divided by 2, and then further divided by both the scan rate and the electrode’s geometric surface area.

Observed in the voltammograms at each step of fabrication is a significant decrease in oxygen reduction after the addition of a silane, maleimide, and aptamer layer on SiO_2_. This is likely due to these additions working to close gaps in the inorganic, creating a more difficult path for oxygen to diffuse through for reduction (Fig. A1a).

Comparing capacitance, bare gold surfaces have a double-layer capacitance around 60 μF/cm^2^ [42]. The addition of 8 nm SiO_2_ significantly reduces the capacitance to 5.99 ± 0.162 μF/cm^2^ and the addition of the silane, maleimide, and aptamer reduces the capacitance further to 2.59 ± 0.160 μF/cm^2^. Comparing this capacitance to thiol-based monolayers have a very similar capacitance to the inorganics as MCH monolayers have a capacitance of 1.671 ± 0.0592 μF/cm^2^ (Fig. A1b).

### A3. Comparison of sensors prepared on gold and 8 nm silicon dioxide

To better compare aptamer performance on oxide films, conventional gold sensors with MCH were also prepared. These sensors were prepared via previously established norms but is also briefly described here. Gold rod electrodes were prepared by polishing in 0.3 and 0.05 μm alumina slurry using a ‘figure eight’ pattern for one minute in each slurry. Next, electrochemical cleaning was performed using CV from −1 to −1.6 V in 0.5 M NaOH and acid cleaning from 0 to 1.6 V in 0.5 M H_2_SO_4_ by 1 volt per second for 300 cycles with a CHI684 potentiostat. Sensors were rinsed in DI H_2_O and functionalized with aptamer by pipetting 25 μL of 500 nM phenylalanine aptamer in 1x PBS on the upward facing gold rod surface and then rested for 1 h at room temperature in darkness. After this, sensors were submerged into 5 mM MCH for at least 12 h (overnight) at room temperature to form the blocking layer surrounding the aptamers.

Through this test, we can see that aptamers deposited onto oxide blocking layers have significantly decreased maximum signal gain at 300 Hz SWV scanning. 8nm SiO_2_ blocking layers enabled a maximum signal gain of ~ 15 – 20 %. In our own tests, with gold sensors, we see > 80 %, leaving a > 60 % gap in signal gain between the two systems (Fig. A2).

### A4. Low resistivity native oxide as working electrode

As was indicated in the main body of the text, thin films of inorganic dielectrics will naturally begin to dissolve from hydrolysis reactions. To evaluate the stability and utility of SiO_2_ films on silicon wafers, low resistivity wafers were used with the native oxide. Low resistivity wafers were used to make the underlying silicon wafer behave as the working electrode replacing gold used previously.

These sensors were prepared by piranha cleaning and then drying as previously described. After cleaning, wafer chips were roughened on the non-polished side with sandpaper and immediately coated with silver epoxy and a conductive wire was affixed to the silver epoxy before drying. Once the epoxy was set, the polished side of the electrode, opposite the silver epoxy connection was covered with Kapton tape with a 3mm hole punched in it. The entire electrode was then sealed on all edges and on unpolished side with hot glue to electrically insulate the sensor. Once the glue was set, electrodes were preliminarily scanned via CV in 1x PBS and then placed into a 1 mM bath of methylene blue suspended in 1x PBS buffer. Scans were then performed at 0.5, 0.1, 0.05, and 0.01 V/s from 0.3 to - 0.8 volts (Fig. A3).

The scans in methylene blue indicate a significant amount of irreversibility in the redox transfer as all scan rates produced peaks that were > 30 mV apart [1]. This would make them unusable for bound redox probes which do not have the ability of diffusion of solution phase redox reporters to the surface. This would also completely negate the use of rapid voltametric techniques like square wave voltammetry which is the gold standard for electrochemical interrogation of sensors. Because of their lack of utility for electrochemical sensors, longevity was not considered further for the native oxide.

### A4. Thermal oxide SiO_2_ on low resistivity silicon wafers

The final experiment considered was to remove any capacitive effects which could have been the result of the thinness of native oxide SiO_2_ layers. These wafers were first prepared by treating low resistivity wafers in hydrofluoric acid then immediately growing 4 nm of thermal oxide on the bare silicon surface. These sensors were then prepared similarly to the other low resistivity electrodes. When placed into 1 mM methylene blue solutions, these electrodes showed no redox transfer, nor any other interfering currents even when the scanning window was expanded to −1 V to 1 V (Fig. A4). Due to the inability of both native and thermal oxide coatings on low resistivity wafers to pass reversible redox transfer, we cannot recommend their use for real time electrochemical monitoring.
